# Efficacy and Safety of Daikenchuto for Constipation and Dose-Dependent Differences in Clinical Effects

**DOI:** 10.1155/2018/1296717

**Published:** 2018-03-05

**Authors:** Tatsuya Hirose, Yasutaka Shinoda, Ayaka Kuroda, Aya Yoshida, Machiko Mitsuoka, Kouki Mori, Yuki Kawachi, Akihiro Moriya, Kouji Tanaka, Atsuko Takeda, Tomoaki Yoshimura, Tadashi Sugiyama

**Affiliations:** ^1^Department of Pharmacy, Ogaki Municipal Hospital, 4-86 Minaminokawa-cho, Ogaki-shi, Gifu 503-8502, Japan; ^2^Laboratory of Pharmacy Practice and Social Science, Gifu Pharmaceutical University, 1-25-4 Daigagu-nishi, Gifu-shi 501-1196, Japan

## Abstract

**Background:**

Daikenchuto (DKT) is a Kampo medicine used for the treatment of constipation. In this study, we evaluated the effectiveness of DKT against constipation.

**Patients and Methods:**

Thirty-three patients administered DKT for constipation were selected and divided into low-dose (7.5 g DKT; *n* = 22) and high-dose (15 g DKT; *n* = 11) groups. We retrospectively evaluated weekly defaecation frequency, side effects, and clinical laboratory data.

**Results:**

Median defaecation frequencies after DKT administration (5, 5.5, 5, and 8 for the first, second, third, and fourth weeks, resp.) were significantly higher than that before DKT administration (2) in all 33 cases (*P* < 0.01). One case (3%) of watery stool, one case of loose stools (3%), and no cases of abdominal pain (0%) were observed. Median defaecation frequencies in the high-dose group (7 and 9) were significantly higher than those in the low-dose group (4 and 3) in the first (*P* = 0.0133) and second (*P* = 0.0101) weeks, respectively. There was no significant change in clinical laboratory values.

**Conclusion:**

We suggest that DKT increases defaecation frequency and is safe for treating constipation.

## 1. Introduction

The prevalence of chronic constipation in the Japanese population is 28% [1]. The global prevalence of chronic constipation is around 14% [2], of which functional constipation accounts for approximately 4.6% [3]. A characteristic symptom of functional constipation is low defaecation frequency and difficulty with bowel movements. Lifestyle habits, bowel habits, and medicine use may induce functional constipation [4]. Functional constipation is caused by neurological or psychological conditions and may be correlated with intestinal inflammation [5, 6].

Lactulose, bisacodyl, polyethylene glycols (PEGs), dietary fiber, and lubiprostone are recommended as treatments for chronic constipation according to the guidelines of World Gastroenterology Organization prepared by Lindberg et al. [7]. However, there is an opinion that the clinical effect of current conventional western medicine, which has a simplified action mechanism, on constipation is limited [8]. These shortcomings can be overcome by using herbal medicines that exert holistic effects to improve functional constipation [9].

Kampo medicine is a Japanese traditional medicine system employing unified herbal combinations [10]. Although there is no description of Kampo medicine in the treatment guidelines for chronic constipation, the use of Kampo medicine in Japan is not uncommon. Daikenchuto (DKT) is the most frequently prescribed Kampo medicine in Japan. It improves blood circulation and enhances peristalsis [11, 12]. It is used for the prevention of postoperative ileus and the treatment of functional constipation [12, 13]. In addition, DKT is used for improving Qi-deficiency and cold; Qi supplied from food and air is one of the several parameters for diagnosis in Kampo medicine. The mechanism underlying these effects of DKT is not found in western medicine, enabling a holistic approach [14]. According to a previous report, DKT improved the self-assessment scale for constipation [15]. However, the examination was conducted only in 10 cases, and stool frequency did not increase.

In addition, there are reports that the quality-of-life (QOL) of constipated patients can be improved by increasing defaecation frequency [16]. The objective of this study was to evaluate the effectiveness of DKT for constipation, with focus on defaecation frequency and dose-related differences in effectiveness.

## 2. Patients and Methods

This study was conducted between January 1, 2013, and December 31, 2015. Thirty-three patients were administered DKT to treat constipation upon admission to Ogaki municipal hospital. Constipation was diagnosed by physicians in the hospital based on ROME III criteria and a defaecation frequency of less than three before DKT administration. DKT was manufactured by Tsumura & Co. (Tokyo, Japan). DKT granules (15 g) contained 10 g of maltose and 1.25 g of a dried extract prepared using 2.0 g of Japanese pepper, 5.0 g of dry processed ginger, and 3.0 g of ginseng radix.

### 2.1. Patients

The exclusion criteria were as follows: cases where the defaecation frequency was four or more one week prior to administration; cases with abnormalities in the gastrointestinal tract; cases in which laxatives were additionally prescribed after DKT administration; cases involving less than seven days of hospitalization before DKT administration; and paediatric cases. There were 33 cases in total during the target period. These cases were divided into low- and high-dose groups. The determination of the therapeutic dose was left at the discretion of the physician. The usual daily dose of DKT is 15 g (6 packs); however, 7.5 g (3 packs) is often used traditionally. There were 22 cases in the low-dose group (7.5 g DKT) and 11 cases in the high-dose group (15 g DKT). Baseline characteristics of all patients enrolled in the study are presented in Table 1.

There were more male patients than female patients among the cases registered. There was no significant difference in patient baseline characteristics between the low- and high-dose groups (Table 1). Data pertaining to cases satisfying the above criteria were extracted from the electronic medical records. Data on aspartate transaminase (AST) and alanine transaminase (ALT) levels, total bilirubin (T-Bil), serum creatinine (Cre), blood urea nitrogen (BUN), Na, K, CL, albumin (Alb), blood sugar (BS), and dietary intake were retrospectively obtained from the electronic medical records.

### 2.2. Effect of DKT on Defaecation Frequency

From the electronic medical records, defaecation frequencies one week before DKT administration (−7 to −1 day) and in the first week of administration (1 to 7 days), second week of administration (8 to 14 days), third week of administration (15 to 21 days), and fourth week of administration (22 to 28 days) were determined and compared before and after DKT administration in the target patients.

### 2.3. Adverse Events Associated with DKT

We investigated side effects of DKT treatment, such as watery stools, abdominal pain, and loose stools, using the electronic medical records of the patients. In addition, changes in clinical laboratory values one week before DKT administration, three days after DKT administration, and one week after DKT administration were compared.

### 2.4. Comparison between Low- and High-Dose Groups

Cases where the daily dose of DKT was 7.5 and 15 g were classified as low-dose and high-dose groups, respectively. From the electronic medical records, defaecation frequencies of the target patients before DKT administration (−7 to −1 day) and in the first week of DKT administration (1 to 7 days), second week of administration (8 to 14 days), third week of administration (15 to 21 days), and fourth week of administration (22 to 28 days) were evaluated and compared between the low- and high-dose groups.

Watery stools, abdominal pain, and loose stools were compared between the low- and high-dose groups. In addition, clinical laboratory values were compared between the low- and high-dose groups before DKT administration and in the first, second, and third weeks of DKT administration.

### 2.5. Sample Size Assessment and Statistical Analysis

We established the following clinically meaningful criteria for sample size calculation: average difference of 3, standard deviation of 4, the probability of *α* error as 0.05, and the probability of *β* error as 0.8. The required number of cases calculated by EZR under these conditions was 28 or more. Statistical analysis was performed using EZR version 1.26. The comparison of median values between the two groups was performed using Mann–Whitney *U* test. For comparing the ratio between the two groups, Fisher's exact probability test was used. In addition, Friedman's test was conducted to compare the median values among the three groups, and the significance level was set to 5%.

### 2.6. Ethical Consideration

The present study was conducted with the approval of the Ethics Committee of the Ogaki municipal hospital [20170727-4].

## 3. Results

### 3.1. Effect of DKT

**Figure 1:**
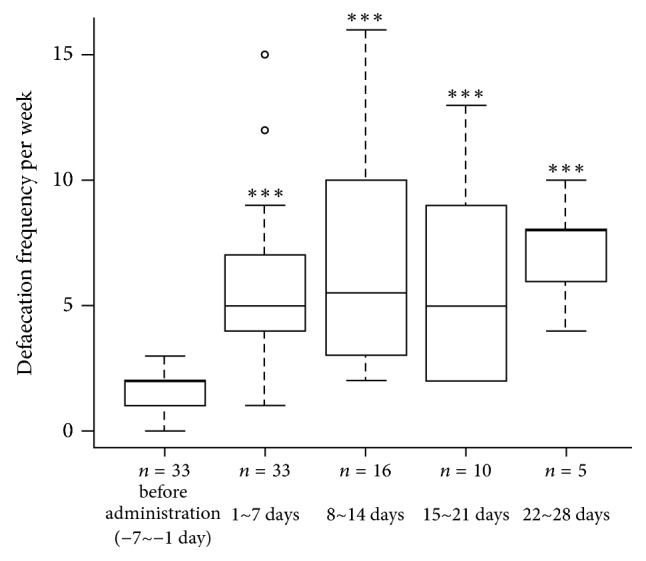
Box plot of defaecation frequency per week before and after Daikenchuto (DKT) administration. The box includes observations from the 25th to 75th percentile, and the horizontal line within the box represents the median value. *∗∗∗* indicates significance compared to values before DKT administration.

In all 33 cases, the median number of defaecations one week (−7 to −1 day) before DKT administration was 2 (interquartile range 1-2). The median number of defaecations in the first (1 to 7 days) and second weeks (8 to 14 days) of DKT administration was 5 (interquartile range 4–7) and 5.5 (interquartile range 3–10), respectively. The median number of defaecations in the third (15 to 21 days) and fourth weeks of DKT administration (22 to 28 days) was 5 (interquartile range 2.25–8.5) and 8 (interquartile range 6–8), respectively, both of which increased significantly (*P* < 0.01) (Figure 1).

The defaecation frequency increased continuously after DKT administration compared with that before DKT administration (Figure 1). In this study, there were no cases using PEG or dietary intervention before DKT administration.

### 3.2. Side Effects of DKT

One case (3%) of watery stool, one case of loose stools (3%), and no cases of abdominal pain (0%) were observed (Table 2). All these side effects improved with continuous DKT administration. There were no significant differences in clinical laboratory values before DKT administration, three days after administration, and one week after DKT administration (Table 3).

### 3.3. Comparison between Low- and High-Dose Groups

**Figure 2:**
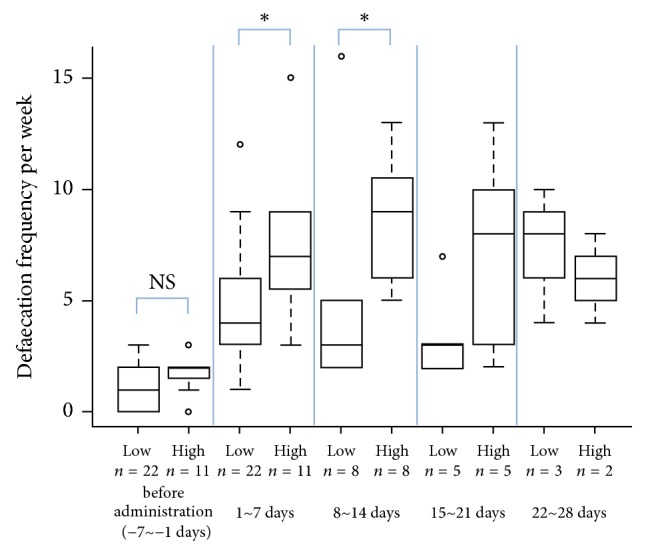
Defaecation frequency per week was compared between the high- and low-dose groups at each week. *∗* indicates significance compared to values between the high- and low-dose groups. NS, not significant.

There was no difference in the number of defaecations before the administration of DKT in the low- and high-dose groups (*P* = 0.364) (Figure 2). In the first week (1 to 7 days) of DKT administration, the median defaecation frequencies in the high- and low-dose groups were 7 (interquartile range 5.5–9) and 4 (interquartile range 3–6), respectively. The defaecation frequency significantly increased in the high-dose group compared to the low-dose group (*P* = 0.0133) (Figure 2). In the second week (8 to 14 days) of DKT administration, median defaecation frequencies in the high- and low-dose groups were 9 (interquartile range 6–10.25) and 3 (interquartile range 2–5), respectively. The defaecation frequency increased significantly in the high-dose group compared to the low-dose group (*P* = 0.0101) (Figure 2).

One case of watery stools was observed in the low-dose group, but there was no difference in side effects between the low- and high-dose groups (*P* = 1) (Table 2). One case of loose stools was found in the high-dose group, but there was no difference in side effects between the low- and high-dose groups (*P* = 0.33) (Table 2).

In the second week of DKT administration (8 to 14 days), Alb values in the low- and high-dose groups was 3.0 (interquartile range 2.6–3.4) and 4.0 (interquartile range 3.8–4.2), respectively. The high-dose group exhibited significantly higher Alb values than the low-dose group (*P* = 0.0317) (Table 4).

## 4. Discussion

Chronic constipation increases the risk of colorectal cancer [17], decreases QOL, and increases medical expenses (US $1,912 to US $7,522 per year) [18]. In this study, we aimed to evaluate the efficacy of DKT in the treatment of constipation and found that DKT can be used as an effective therapeutic agent for constipation.

DKT has been reported to be effective against constipation in specific cases, such as Parkinson's disease, pregnant women, and stroke patients [19–21]. Yuki et al. reported that DKT significantly improves constipation and indigestion in chronic constipation based on the Gastrointestinal Symptom Rating Scale [15]. Thus, it can be suggested that DKT significantly improves constipation and self-evaluated indigestion. This previous study reported that there was no increase in the number of defaecations; moreover, only 10 patients were included in the study. Another study reported that DKT had no effect on functional constipation in women [22]. However, our results contradict these previous reports. This could be because 72.7% of the patients in our study were males.

The increase in defaecation frequency relative to functional constipation has not been confirmed in previous studies. In the present study, DKT increased defaecation frequency, and the effect was sustained up to four weeks after administration.

To our knowledge, this is the first study to report the increase in defaecation frequency following DKT administration in patients with functional constipation. DKT acts on the intestinal smooth muscle cells and accelerates motilin secretion, activates transient receptor potential cation channel subfamily V member 1 and transient receptor potential cation channel subfamily A member 1, and inhibits potassium two-pore domain channel subfamily K member 9 channel, thereby promoting intestinal motility [23–26]. In addition, it has been reported that DKT enhances intestinal motility and promotes the transport of intestinal contents in both animals and healthy adults [27, 28]. In this study, there were no cases of PEG or dietary fiber intake before DKT administration, and the influence of other drugs on defaecation frequency was considered to be small.

It was thought that DKT promoted peristalsis in the intestine to increase defaecation frequency via the action mechanisms described above. Moreover, the subjects in this study were elderly, and they may show reduced intestinal peristalsis. This suggests that DKT may increase defaecation frequency by promoting peristaltic movement in the intestinal tract.

DKT promotes the secretion of calcitonin gene-related peptide and adrenomedullin via the transient receptor potassium channels [11, 25]. In addition, it increases intestinal blood flow [11]. These mechanisms have not been described in western medicine, and it is considered that these mechanisms contribute to holistic effects of DKT and enhances constipation treatment. The high-dose group showed increased defaecation frequency compared to the low-dose group. Moreover, the side effects observed in the high-dose group were comparable to those observed in the low-dose group. These findings suggest that a high dose of DKT is more effective than a low dose. Therefore, a high dose could be recommended if an enhanced effect is desired. Nevertheless, treatment of constipation is possible at low doses.

From the electronic medical records, side effects associated with DKT administration were watery stool (one case; 3%) and loose stools (one case; 3%); no case of abdominal pain was reported. Because these side effects improved upon the continuous administration of DKT, they were considered minor side effects. Senna (sennoside) is used as a medicine for various purposes worldwide, including the treatment of constipation. However, it has been reported to cause diarrhoea (82.8%) and abdominal pain (70.4%) [29, 30].

Therefore, the long-term use of laxatives, including sennoside, might increase the frequency of abdominal pain and diarrhoea and impair the QOL of patients. Nevertheless, as DKT did not cause abdominal pain or diarrhoea, it may be considered safe without affecting the QOL of patients.

The high-dose group exhibited significantly higher Alb values than the low-dose group in the second week of DKT administration. This is an important difference as some drugs are transported in the Alb-bound form. However, this cannot be directly considered as the effect of drugs. The fluctuation in clinical laboratory values following DKT administration was small and this change had no clinical significance. On the other hand, it may be due to the improvement in nutritional status with the alleviation of constipation. DKT may be considered as a safe and effective treatment for constipation.

The present study has several limitations. First, this was a retrospective study and QOL evaluation was not performed. Second, owing to the small sample size, the risk of bias is high, and results are not conclusive. Third, the effects of DKT were not compared with other medications for constipation. Fourth, since cases in which laxatives were additionally prescribed after DKT administration were excluded, there is a possibility that more severe cases were treated with two or more medications and therefore not included in the analysed group. Fifth, we could not investigate the lot number of DKT. Therefore, there is a possibility that the effects and safety differed depending on the lot number of DKT administered. Based on these results, with great cautiousness, we suggest that DKT may be effective and safe to treat constipation. In the future, it is necessary to confirm the benefits of DKT for treating constipation by conducting a prospective randomised placebo comparison study.

## Figures and Tables

**Table 1:** Baseline characteristics of patients.

Characteristics	Total (*n* = 33)	Subgroups		*P* value
		Low-dose group (*n* = 22)	High-dose group (*n* = 11)	
Male	24 (72.7%)	16 (72.7%)	8 (72.7%)	1
Age, years	74 (68–80)	75 (63–80)	74 (72–80)	0.154
Height, cm	161 (155–166)	162 (155–165)	161 (153–166)	0.924
Weight, kg	57 (50–63)	58 (52–64)	52 (44–61)	0.194
BMI, kg/m^2^	22 (18–25)	22 (19–25)	20 (17–24)	0.154
Duration of DKT administration, days	12 (7–20)	8 (5–15)	18 (10–24)	0.075
Percentage of dietary intake eaten to fed before DKT administration, %	60 (50–90)	60 (50–85)	70 (50–85)	0.671
Time since last bowel movement, days	4 (2–6)	4 (2–6)	5 (3–6)	0.714
Concomitant drugs				
Heavy magnesium oxide	21 (63.6%)	14 (63.6%)	7 (63.6%)	1
Senna	17 (51.5%)	10 (45.5%)	7 (36.6%)	0.3245
Opioids	4 (12.1%)	3 (13.6%)	1 (9.0%)	0.7061
Chemotherapy	4 (12.1%)	2 (9.0%)	2 (18.2%)	0.4507
Complications				
Parkinson's disease	0 (0%)	0 (0%)	0 (0%)	1
Cerebral vascular disease	5 (15.2%)	5 (22.7%)	0 (0%)	0.0861
Diabetes	8 (24.2%)	5 (22.7%)	3 (27.3%)	0.7739
Laboratory data				
Na (mEq/L)	139 (136–141)	139 (136–141)	141 (138–142)	0.273
K (mEq/L)	4.2 (4.0–4.5)	4.2 (3.9–4.4)	4.4 (4.1–4.5)	0.893
Cl (mEq/L)	102 (100–105)	103 (99–105)	102 (101–104)	0.863
AST (IU/L)	20 (18–24)	20 (18–24)	20 (18–25)	0.968
ALT (IU/L)	16 (12–23)	18 (12–26)	14 (10–18)	0.0874
T-Bil (mg/dL)	0.6 (0.4–0.7)	0.5 (0.4–0.8)	0.6 (0.41–0.7)	0.809
BUN (mg/dL)	16 (14–20)	17 (14–22)	15 (13–16)	0.231
Cre (mg/dL)	0.8 (0.6–1.0)	0.8 (0.6–1.0)	0.9 (0.6–1.0)	0.633
Alb (g/dL)	3.4 (3.3–3.7)	3.4 (3.3–3.8)	3.4 (3.2–3.7)	0.901
BS (mg/dL)	112 (97–137)	115 (102–137)	109 (97–141)	1

AST, aspartate aminotransferase; ALT, alanine aminotransferase; T-Bil, total bilirubin; Cre, serum creatinine; BUN, blood urea nitrogen; Alb, albumin; BS, blood sugar; DKT, Daikenchuto.

**Table 2:** Side effects of Daikenchuto administration.

Adverse event	Total (*n* = 33)	Subgroups		*P* value
		Low-dose group (*n* = 22)	High-dose group (*n* = 11)	
Watery stools	1 (3%)	1 (4.5%)	0	1
Abdominal pain	0	0	0	1
Loose stools	1 (3%)	0	1 (9%)	0.33

**Table 3:** Clinical laboratory data of patients.

	Prior to DKT administration	Three days after DKT administration	One week after DKT administration	*P* value
Na (mEq/L)	139 (136–141)	137 (134–140)	139 (137–140)	0.318
K (mEq/L)	4.2 (4.0–4.5)	4.2 (3.8–4.6)	4.4 (3.9–4.7)	0.0657
Cl (mEq/L)	102 (100–105)	101 (99–105)	102 (99–105)	0.123
AST (IU/L)	20 (18–24)	18 (13–28)	21 (16–27)	0.927
ALT (IU/L)	16 (12–23)	13 (11–23)	18 (11–25)	0.513
T-Bil (mg/dL)	0.6 (0.4–0.7)	0.5 (0.3–0.7)	0.5 (0.4–0.7)	0.284
BUN (mg/dL)	16 (14–21)	19 (15–26)	15 (10–24)	0.368
Cre (mg/dL)	0.82 (0.62–1.0)	0.7 (0.6–1.0)	0.7 (0.6–0.9)	0.744
Alb (g/dL)	3.4 (3.3–3.7)	3.2 (2.7–3.8)	3.2 (2.9–3.5)	0.135
BS (mg/dL)	112 (97–137)	101 (91–134)	105 (98–119)	0.607

AST, aspartate aminotransferase; ALT, alanine aminotransferase; T-Bil, total bilirubin; Cre, serum creatinine; BUN, blood urea nitrogen; Alb, albumin; BS, blood sugar; DKT, Daikenchuto.

**Table 4:** Clinical laboratory data in the high- and low-dose groups.

	First week of DKT administration		*P*-value	Second week of DKT administration		*P* value	Third week of DKT administration		*P* value
	Low-dose group (*n* = 22)	High-dose group (*n* = 11)		Low-dose group (*n* = 8)	High-dose group (*n* = 8)		Low-dose group (*n* = 5)	High-dose group (*n* = 5)	
Na (mEq/L)	139 (137–140)	139 (137–141)	0.938	139 (133–141)	137 (134–142)	0.792	136 (132–137)	138 (137–139)	0.291
K (mEq/L)	4.3 (4.0–4.5)	4.5 (3.9–4.9)	0.615	4.3 (3.8–4.6)	4.6 (4.3–4.8)	0.269	4.7 (4.2–4.9)	4.6 (4–5.5)	1
Cl (mEq/L)	103 (98–105)	101 (100–105)	0.938	103 (99–105)	104 (102–105)	0.792	100 (98–102)	102 (96–105)	0.786
AST (IU/L)	23 (18–35)	17 (15–21)	0.153	24 (19–34)	17 (15–23)	0.131	21 (20–25)	15 (14–18)	0.0668
ALT (IU/L)	21 (13–27)	13 (11–21)	0.315	17 (13–26)	16 (10–18)	0.487	12 (12–18)	12 (12–18)	1
T-Bil (mg/dL)	0.6 (0.4–0.8)	0.5 (0.4–0.5)	0.326	0.5 (0.4–0.5)	0.4 (0.3–0.5)	0.283	0.6 (0.6–0.6)	0.7 (0.5–0.8)	1
BUN (mg/dL)	21 (13–24)	11 (10–14)	0.142	17 (15–26)	18 (14–24)	0.779	12 (10–13)	18 (12–26)	0.25
Cre (mg/dL)	0.7 (0.6–1.0)	0.8 (0.6–0.9)	0.91	0.6 (0.5–0.7)	0.7 (0.6–0.9)	0.461	0.5 (0.4–0.6)	0.7 (0.6–0.9)	0.393
Alb (g/dL)	3.2 (2.5–3.3)	3.3 (3.0–4.0)	0.443	3.0 (2.6–3.4)	4 (3.8–4.2)	0.0317	3.0 (3.0–3.2)	3.8 (3.8–4.4)	0.0722
BS (mg/dL)	108 (101–121)	99 (98–114)	0.69	133 (125–140)	106 (99–111)	0.2	89 (89-89)	100 (97–102)	0.667

AST, aspartate aminotransferase; ALT, alanine aminotransferase; T-Bil, total bilirubin; Cre, serum creatinine; BUN, blood urea nitrogen; Alb, albumin; BS, blood sugar; DKT, Daikenchuto.
